# Hen’s egg ladder: Therapy option for the gradual introduction of hen’s eggs in cases of hen’s egg allergy 

**DOI:** 10.5414/ALX02517E

**Published:** 2024-10-16

**Authors:** Amely Brückner, Petra Funk-Wentzel, Stephanie Hompes

**Affiliations:** 1 Elbe Klinikum Buxtehude, Clinic for Dermatology, Competence Center for Chronic Skin Diseases, Buxtehude,; 2 Practice for Nutrition Therapy, Stuttgart, and; 3 Altona Children’s Hospital, Hamburg, Germany

**Keywords:** hen’s egg allergy, egg ladder, food ladder, step-by-step plan for introduction of hen’s egg, baked egg products, individual tolerance, acquisition of tolerance, patient selection

## Abstract

More than 10 years ago, the British Society for Allergy and Clinical Immunology (BSACI) published guidelines for the management of egg allergy [1]. For the first time, these included a stepwise plan for the reintroduction of egg for egg-allergic children who could already tolerate well-cooked egg, such as cakes and cookies. Since then, various egg ladders have been developed [2, 3, 4, 5, 6, 7, 8, 9]. In the past 3 years, several studies have been published suggesting that a gradual introduction of highly processed to less processed egg containing foods contribute to the acceleration of tolerance development [2, 3, 4, 5]. However, depending on the study and egg ladder, the egg products vary in their level of processing (wheat matrix, degree, and location of heating (e.g., oven, pan, pot), egg quantity, and egg protein). In the UK, the introduction of the egg ladder is recommended at the age of 12 months or if the last reaction occurred 6 months before. The benefits of introducing egg at home include an early increase in the variety of foods, reduction of food fears, improved nutrient intake, and the avoidance of hospitalization fears in children [10]. Children with mild reactions in the past can start with small amounts of baked goods at home. Food challenges in an inpatient setting to exclude or reconfirm the allergy should be conducted if the patients have previously had severe allergic reactions, i.e., anaphylaxis, or if the smallest amounts triggered an allergic reaction or if existing asthma is poorly controlled [10, 11]. The present work includes, in addition to the evaluation of study results, a presentation of the recent studies regarding egg ladders. From these, a new egg ladder as therapeutic option for the German-speaking region has been developed. As already done for the milk ladder a detailed step-by-step plan, selection criteria, a recipe collection, and also ideas for commercial prepackaged food items can be found in the appendices [11].

## Introduction: Hen’s egg ladder as a treatment option for hen’s egg allergy 

More than 10 years ago, the British professional association BSACI published guidelines on the management of egg allergy [1]. These included, for the first time, a step-by-step plan for the reintroduction of hen’s egg to children who are allergic to egg and who already tolerated well-cooked egg, e.g. in cakes and biscuits, very well. Since then, various “egg ladders” have been developed [2, 3, 4, 5, 6, 7, 8, 9]. In the last 3 years, various studies have been published suggesting that a gradual introduction of highly processed to less processed egg products helps to accelerate tolerability [2, 3, 4, 5]. However, depending on the study and the “egg ladder”, the foods containing hen’s egg vary in terms of their processing (wheat matrix, degree and method of heating (e.g., oven, pan, pot), amount of hen’s egg, and hen’s egg protein). 

In the UK, the introduction of baked hen’s eggs after a relatively mild cutaneous reaction to a significant trigger quantity (e.g., a portion of scrambled eggs), is recommended as a possible first step on the egg ladder at the age of 12 months with the last reaction occurring 6 months previously [10]. According to BSACI guidelines, the introduction of baked hen’s eggs improves quality of life and eases restrictive diets [10]. The authors see further advantages of reintroducing hen’s eggs at home by means of a structured step-by-step plan in an early increase in food variety and thus an improved nutrient supply. In addition, children’s fears of food are reduced and fears of hospitalization are avoided [10, 11]. Children with mild reactions in the past can start with small amounts of baked goods at home. Food challenges under inpatient conditions to exclude or reconfirm the allergy should be performed if the patient has previously had a severe allergic reaction, i.e., anaphylactic reaction, or if the smallest quantities have triggered the allergic reaction or if existing asthma is poorly controlled [10, 12]. 

So-called “food ladders” are not oral immunotherapy (OIT) and should be clearly distinguished from it. OIT, which in Germany is only approved for the treatment of primary peanut allergy, is a treatment option for persistent food allergy [13, 14]. The initiation of an OIT and any increase in dosage must be carried out under medical supervision [13]. The aim of OIT is to increase the threshold dose for triggering an allergic reaction so that no allergic symptoms develop despite unintentional consumption of very small amounts of the allergen [15]. In contrast, the use of food ladders in the home environment is best carried out until complete tolerance is reached [10, 15]. 

The development of the hen’s egg ladder presented here as a therapeutic option for German-speaking countries is based on the evaluation of study results and assessment of the existing hen’s egg ladders. As already done for the milk ladder [12], a detailed step-by-step plan, selection criteria, a collection of recipes and – newly added here – a selection of commercially available, packaged foods can be found in the appendix . 

## Prevalence and prognosis of hen’s egg allergy 

Alongside allergies to cow’s milk and peanuts, hen’s egg allergy is one of the most common food allergies in childhood [14, 16]. The pooled prevalence for all age groups of self-reported hen’s egg allergy in Europe is between 1.8% and 3.0% [17]. In the EuroPrevall birth cohort, the incidence of hen’s egg allergy confirmed by food challenge was just under 9% at 2 years of age [16]. About 2/3 of all children with a positive oral provocation test for hen’s eggs had atopic dermatitis [18]. In adults, hen’s egg allergy is considered rare at 0.1% [19]. Data from the anaphylaxis register show that hen’s eggs are one of the most common triggers of anaphylaxis in the first 2 years of life [20]. 

In addition to rapidly occurring IgE-mediated symptoms, hen’s egg allergy can also manifest as a non-IgE-mediated allergy with delayed symptoms of atopic eczema [21]. 

The prognosis for tolerance development is generally favorable. Approximately 50% of patients become tolerant within 12 months of diagnosis [16]. By the age of 16, at the latest, ~ 70 – 80% of hen’s egg allergy sufferers can consume hen’s egg in any form of preparation [21, 22, 23]. Various studies show that the prognosis for later tolerance is particularly good if baked hen’s egg products are already tolerated or regularly consumed [24, 25, 26]. On the other hand, data from the HealthNuts cohort showed that of the children who could not tolerate baked egg at 1 year of age, only 13% had become tolerant (24). Persistent hen’s egg allergy appears to be associated with more severe allergic reactions to baked hen’s egg at 12 months of age, with IgE antibody levels ≥ 50 kU/L and with the presence of other food allergies and/or atopic comorbidities [23, 27]. 

However, various studies also show that between 53% and 63% of egg-allergy sufferers tolerated baked hen’s egg products despite having experienced anaphylaxis to hen’s eggs, depending on the study [24, 28, 29, 30]. 

## Hen’s egg allergens and their characteristics 

The most important hen’s egg allergens are ovomucoid (Gal d 1) and ovalbumin (Gal d 2). The heat-labile and digestion-labile ovalbumin is the most abundant protein in egg white. The glycoprotein ovomucoid, on the other hand, is considered the most allergenic due to its heat and digestion stability resulting from numerous disulfide bridges. It remains soluble even after boiling for 1 hour and is therefore allergologically relevant [25, 26]. 

On the other hand, immunoblots have shown that ovomucoid baked into wheat matrix significantly loses allergenicity [33, 34]. The Dutch study by De Vlieger et al. [3] was able to demonstrate in the application of the hen’s egg ladder that the IgE binding of ovalbumin greatly reduced by heating, but not initially in the case of ovomucoid. Only when heated in combination with wheat proteins could a reduced binding capacity for ovalbumin and ovomucoid be determined in this study, which is also consistent with earlier observations [3, 33, 34, 35]. 

**Figure 1. Figure1:**
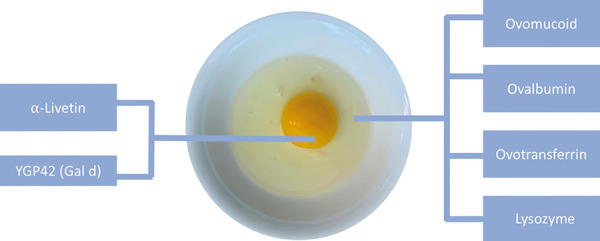
Hen’s egg allergens in the egg yolk (left) and in the egg white (right) [31, 32]. Graphic/Image: Amely Brückner.

It is assumed that both the dough components and the intensive mixing as well as the thermal influence of the baking process cause a high stability of the cereal structure and thus delay allergen release through mechanical and enzymatic activity [36]. [Fig Figure1]

## International use of hen’s egg ladders and assessment of safety by study teams 

The generally good tolerance of baked goods containing hen’s eggs and the acceleration of tolerance development with their regular administration seem to have been groundbreaking for the development of hen’s egg ladders [25, 37]. In the meantime, various working groups have tested the use of hen’s egg ladders. 

In Newcastle, Australia, a 6-step hen’s egg ladder was started in 2018 in patients aged 1 – 5 years, provided there was no previous anaphylaxis. Some started with a tolerance for baked egg, but overall, it took them 9 – 21.5 months to climb the ladder. Parental satisfaction was very high. In rare cases, there were mild reactions; in 1 case, the adrenaline auto-injector was used on a 6-year-old child when moving to stage 6, i.e., when raw hen’s eggs was eaten. The authors consider the hen’s egg ladder to be safe if patients are carefully selected [5]. 

In the Israeli study by Gotesdyner et al. [2], an intervention group (children < 2 years) using a graduated protocol for the reintroduction of hen’ eggs was compared with a control group with strict avoidance of hen’s eggs. It was shown that in the intervention group, which already tolerated baked hen’s eggs, hen’s eggs could be safely introduced by means of a graduated protocol over 3 stages and tolerance was achieved ~ 2 years earlier than in the avoidance group. Early introduction of hen’s eggs using a ladder should be the responsibility of trained and experienced allergy teams [2]. 

The 4-step milk and egg ladder from Canada, published in 2021, was reviewed for safety and effectiveness. 79 patients with hen’s egg allergy completed the egg ladder, 16 of whom climbed the milk ladder at the same time. The age of the participating egg allergy sufferers ranged from 7 months to 15 years, with the average age of just over 3 years. At the beginning of the study as well as after 3, 6, and 12 months the progression was surveyed. At baseline, over 70% were already able to tolerate some form of hen’s egg preparation, predominantly baked into muffins. Over the course of the study, an increasing number of patients were able to tolerate less processed hen’s egg preparations. Skin symptoms such as urticaria were the most common side effect, while adrenaline auto-injectors as an indicator of anaphylaxis only had to be used twice in this cohort (in a total of 109 children), with both children being over 6 years old (9 and 14 years old). The authors therefore recommend establishing selection criteria for participation in a food ladder, including only younger children [38]. 

When carrying out the 3-stage egg ladder in Ireland according to the IFAN scheme, a distinction is made between “well cooked”, “less well cooked”, and “almost raw”. The individual stages themselves include further steps with increasingly less processing and at increasing amounts of hen’s egg. When using this ladder in the study by Cotter et al. [4], the development of tolerance was tested in 29 children after 6 and 12 months. On average, the children needed 8 months to tolerate “almost raw” egg products; ~ 1/3 achieved this within 6 months and 2/3 within a year. Two children made no progress within 6 or 12 months, respectively, which was explained by the parents’ reluctance. Children with other food allergies took longer to develop a tolerance to hen’s egg. However, other atopic diseases played no role in the development of tolerance. One child required adrenaline due to anaphylaxis caused by the early accidental consumption of a raw egg product (meringue). This child continued on the ladder and became completely egg-tolerant after another 4 months. The group of authors led by Hourihane declares the IFAN ladder to be safe and effective for most egg allergy sufferers and has almost completely discontinued oral food challenges with hen’s egg [4]. 

One of the most recent studies, also from Ireland, re-examined the development of tolerance in children allergic to cow’s milk and hen’s egg, particularly in relation to a history of anaphylaxis to the respective food. This study included 287 hen’s egg allergy sufferers under the age of 3 years. They completed the hen’s egg ladder according to the IFAN scheme mentioned above. On average, the children needed just over 23 months to reach the tolerance target of > 30 g raw egg (in the form of meringue, mayonnaise, or other). Children with previous anaphylaxis were more likely to have allergic reactions as the ladder progressed than children without anaphylaxis (35.7 vs. 25.4%). These reactions in particular are associated with lower chances of tolerance. Nevertheless, more than 85% of children who had reported anaphylactic reactions prior to starting the hen’s egg ladder achieved full hen’s egg tolerance compared to 93% who had not previously shown anaphylaxis [39]. 

The Dutch study by De Vlieger et al. [3] mentioned above also examined the development of tolerance using the hen’s egg ladder. 78 people over the age of 1 year who were already tolerant to baked goods were divided into two arms and observed over different periods of time (over a total of 24 vs. 30 months). In contrast to other hen’s egg ladders, the order differed in that the hard-boiled egg was given at the second stage after cake in this ladder. 58 children (74%) tolerated raw hen’s eggs at the end of the study, whereby the group with the shorter introduction time was slightly superior to the other group. The authors consider the implementation of this hen’s egg ladder to be safe, as no severe allergic reactions or eosinophilic esophagitis occurred [3]. 

Conclusion: The gradual climbing of hen’s egg ladders is described in all the studies presented as an effective way to develop tolerance when patients are specifically selected. 

## Recommended procedure for using the presented hen’s egg ladder 

The following new hen’s egg ladder for German-speaking countries with its various steps was developed on the basis of scientific findings regarding the altered allergenicity of hen’s eggs during processing, heating, and the so-called matrix effects. The individual steps are also based on practicability and on the experiences already gained by specialized allergy nutritionists in dialogue with their patients and their caregivers. 

The presented hen’s egg ladder consists of the following steps:

- Pastries/bread
- Dried egg pasta
- Pancakes
- Meatballs/vegetable patties
- Pure hen’s egg: hard-boiled – soft-boiled – scrambled/fried egg
- Optional: Food preparations with raw hen’s egg

Like all available hen’s egg ladders, the hen’s egg ladder presented here starts with baked goods in which small amounts of hen’s egg (1 – 2 eggs) are baked in a large amount of wheat (~ 500 g) over a longer period of time. It is important that the baked goods are very well baked inside. Therefore, either small portion sizes (such as cookies, muffins) or very long baking times (as with bread) are recommended. Sweet and savory variations provide variety and, as they can be easily portioned, you can start with very small amounts of hen’s egg. 

The progression up the hen’s egg ladder is always based on individual tolerance. Preparations with raw hen’s egg at the end of the ladder are only optionally recommended, as raw hen’s eggs should not be offered to small children anyway due to the risk of infectious diseases [40]. However, the authors’ experience shows that licking cake batter or eating desserts and ice cream containing raw hen’s eggs can certainly happen. If level 6 is reached, the use of pasteurized hen’s eggs should be advised. 

Patients and their parents who are eligible for the hen’s egg ladder should be informed in detail about the objectives, procedure, possible risks, and benefits. The aim is to introduce compatible hen’s egg preparations and thus a greater variety of foods. After the information has been provided, the decision should first be made by the patient’s parents. The implementation of the individual steps of the step-by-step plan should be documented. If there are any questions or problems, it should be possible to contact a doctor and/or nutritionist. Regular check-ups are recommended [12]. 

Appendices 1 – 3 present the 6-step hen’s egg ladder for introducing hen’s egg products with recipes for practical application. The hen’s egg ladder can also be successfully implemented with commercial, packaged foods if the appropriate products are selected by checking the ingredients list and nutritional table. Not suitable are baked goods that are either not well baked and therefore contain insufficiently heated hen’s eggs (“sludge cakes”) or baked goods that contain large quantities of hen’s eggs for binding, such as cheesecake with cream cheese and hen’s eggs. 

The procedure according to the hen’s egg ladder represents a framework plan. Deviations such as a faster or slower approach as well as smaller or larger consumption quantities can be individually adapted. Regardless of the introduction of hen’s eggs, a balanced, healthy, and needs-oriented diet should be ensured. 

An experienced nutritionist should accompany the use of the hen’s egg ladder in terms of nutritional therapy. 

## Safety aspects when using the presented hen’s egg ladder 

The favorable prognosis of a hen’s egg allergy should not obscure the fact that hen’s egg-allergic patients can experience severe allergic reactions. Therefore, special safety considerations apply to the introduction of hen’s egg in a home setting, based on those of the milk ladder [12]:

- The age of the patients should not exceed preschool age. The younger the better!
- No asthma is ideal or existing asthma should be well controlled.
- No patients with a history of anaphylaxis with respiratory or cardiovascular symptoms in temporal relation to hen’s egg consumption.
- In case of use in IgE-mediated hen’s egg allergy, no low threshold dose should have triggered the reaction.
- Parents should be able to recognize emergency situations, administer emergency medication if necessary, and call for help quickly.
- The patient and therefore also the parents as carers must be adherent. Individual tolerance can vary greatly from patient to patient as the treatment progresses.
- Parents must be able to recognize when a dose increase is possible, should be temporarily suspended, or delayed.
- Atopic eczema and other underlying diseases should be under control.
- There should be as few communication and language barriers as possible between the families and the counsellors.

Even though Gotesdyner et al. [2] were able to show that even patients who had already experienced anaphylaxis to hen’s eggs benefited from a gradual reintroduction of hen’s egg in terms of a more rapid development of tolerance [2], introduction is not recommended for these patients. If this option is nevertheless considered, these patients should be given special consideration as to whether and under what circumstances the introduction of hen’s egg in the home environment is suitable. Carrying an emergency kit and practicing emergency management is essential for these patients [4]. 

In any case, it is recommended to have antihistamines on hand in order to be able to intervene early in the event of an allergic reaction [10]. 

For simple practical application, it is advisable to use the 4 A’s (age, asthma, anaphylaxis, adherence) as a guide [41]. 

Conclusion: After careful patient selection, taking into account the above criteria, the implementation of the hen’s egg ladder may be a suitable method for the introduction of hen’s egg. [Table Table1]

## Authors’ contributions 

A. Brückner, P. Funk-Wentzel, and S. Hompes contributed to the development of the step-wise plan of the hen’s ladder, the calculations and recipe creations. A. Brückner wrote the paper with the input from all authors. 

## Funding 

None. 

## Conflict of interest 

The authors declare that there is no conflict of interest with regards to this publication. 

**Table. Table1:** Checklist for food ladders.

**Which children are probably NOT suitable for the milk or egg ladder? The 4 A’s**
□ **Age:** Children ≥ 6 years old
□ **Asthma:** Severe or poorly controlled asthma (≥1 criteria met):
○ Emergency admission or hospitalization (for asthma) within the last 6 months
○ Need to take oral corticosteroids in the last 6 months
○ Nocturnal symptoms, especially use of salbutamol spray
○ Asthma symptoms on at least 3 days within 1 week
○ Need for 3 or more doses of emergency (salbutamol) spray every week
○ Absence from nursery or school due to asthma in the last 3 months
○ No usual sport or age-appropriate everyday activity (“keeping up with peers”) due to asthma symptoms
□ **Anaphylaxis**: History of allergic reaction to very small amounts of the relevant food, especially in the case of baked products
□ **Insufficient adherence**: The patient or caregivers are not able to adhere to the necessary instructions and measures for the hen’s egg ladder (e.g., time management, language barrier).

If any of the above points apply, the hen’s egg ladder should not be performed [33] (adapted from Chua et al. [41]).

## Supplemental material

Supplemental material Appendix I – IV.
