# Supplementary material for: Hen’s egg ladder: Therapy option for the gradual introduction of hen’s eggs in cases of hen’s egg allergy
Source: Allergol Select. 2024 Oct 16;8:324–31. doi: 10.5414/ALX02517E (PMC11500597; doi:10.5414/ALX02517E)
Supplement: Supplemental material [file allergologieselect-8-324-S01.pdf]

Appendix I

Hen's egg ladder = step-by-step plan for hen's egg introduction<br>developed 5/2024 by Amely Brückner, Petra Funk-Wentzel, Stephanie Hompes					
6th level (optional)	Preparations with raw hen's egg<br>including raw cake dough, sorbet with beaten egg whites, meringue, Bavarian cream, Tiramisu, Mousse au Chocolat, Frosting , Sugar-Egg Glaze , etc.				
5th level	Pure hen's egg – differences in cooking time<br>Scrambled eggs/fried eggs<br>↑<br>boiled until soft (5 – 6 min.)<br>↑<br>hard boiled (10 – 15 min.)	**Amount**		**Hen's egg content**	**Protein content on average**
		1 hen's egg		56 g	7.3 g
		½ hen's egg		28 g	3.6 g
		¼ hen's egg		14 g	1.8 g
		1/8 hen's egg		7 g	0.9 g
4th level	**Meatballs/Vegetable patties**<br>Hen's egg as a binding agent, well cooked				
		1 vegetable patty (67 g)		14 g	1.8 g
		½ vegetable patty (34 g)		7 g	0.9 g
		¼ vegetable patty (17 g)		3.5 g	0.45 g
		1/8 vegetable patty (8.5 g)		1.8 g	0.23 g
		1 meatball (55 g)		4.7 g	0.6 g
		½ meatball (27 g)		2.4 g	0.3 g
		¼ meatball (14 g)		1.1 g	0.15 g
3rd level	**Pancakes**<br>Hen's egg baked with flour, shorter baking time				
		1 pancake		9.4 g	1.21 g
		½ pancake		4.7 g	0.61 g
		¼ pancake		2.3 g	0.30 g
		1/8 pancake		1.2 g	0.15 g
2nd stage	**Dried egg pasta**<br>with hen's egg content 10%<br>Cooked for 8 – 15 minutes				
		50 g cooked pasta		1.7 g	0.2 g
		2 tbsp (20 g) cooked pasta		0.6 g	86 mg
		1 tbsp (10 g) cooked pasta		0.3 g	43 mg
		1 tsp (5 g) cooked noodles		0.2 g	22 mg
1st stage	**Pastries, bread**<br>Bake small amounts of hen's egg with flour	1 muffin[2]	2 slices of bread[4]	~ 10 g	1.3 g
		1 muffin[1]	2 slices of bread[3]	~ 4.5 g	~ 0.6 g
		½ muffin[1]	1 slice of bread[3]	~ 2.3 g	~ 0.3 g
		¼ muffin[1]	½ slice of bread[3]	~ 1.15 g	~ 0.15 g
		1/8 muffin[1]	¼ stalk of bread[3]	~ 0.58 g	~ 70 mg
Start		1/16 muffins[1]	1/8 cup bread[3]	~ 0.29 g	~ 40 mg
		1/32 muffins[1]	1/16 Sch. B.[3]	~ 0.15 g	~ 20 mg

[1]Muffin dough with 1 egg (size M ~ 55 g) baked for 12 pieces; [2]muffin dough with 2 eggs (size M 55 g each) baked for 12 pieces; [3]bread dough with 1 egg (size M ~ 55 g) for 24 slices; [4]bread dough with 2 eggs (size M ~ 55 g) for 24 slices.

Purchased, packaged foods can also be used. Checking ingredient lists and nutritional tables is essential (see "Risk ramp").

Baked goods that are either not baked well and therefore contain insufficiently heated hen's eggs (slush cakes) or baked goods that contain large amounts of hen's eggs for binding, such as cheesecakes with quark mixture containing hen's eggs, are not suitable.

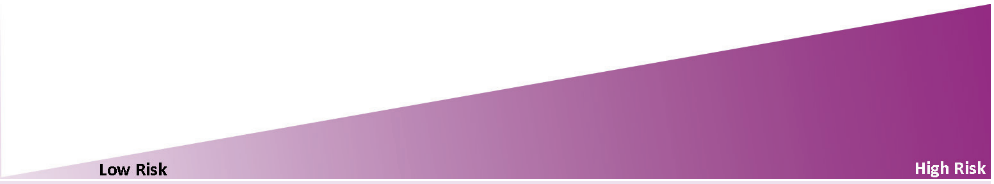

Baked in the oven		Cooked	Baked/fried		Cooked/ fried	Raw (pasteurized)
Bread with 1 egg<br><br>Muffin with 1 egg	Bread with 2 eggs<br><br>Muffin with 2 eggs	Egg noodles	Pancakes	Meatballs/ Vegetable patties	Boiled egg<br><br>Scrambled eggs<br><br>Fried egg	Raw cake dough, sorbet

"Risk ramp": The less egg and the higher the processing (thermal heating, baking time and wheat matrix), the lower the risk of reaction.

Appendix II

Recipes (by Amely Brückner, Petra Funk-Wentzel, and Stephanie Hompes)

Notes on the application of the step-by-step plan:

- An allergological check-up is necessary before starting!
- Before you start the introduction or switch to the next stage, you should always be sure that your child is well and has no illness.
- As a rule, start with level 1 (bread or muffins). If your child is already eating the foods in this level, start with the next level.
- When you start the step-by-step plan or apply the next step, please ensure that you have your child under observation for at least 2 hours after eating the new food.
- The same quantity/preparation should always be consumed on 3 days within a week without causing symptoms before the quantity or preparation is increased.
- Only switch to the next level when your child has already eaten the food from the current level several times without symptoms, preferably in the largest amount listed or in an age-appropriate amount. The speed of the increase can vary greatly form child to child
- Once the egg has been successfully introduced in one stage, the food in question, e. g., muffins, should continue to be consumed several times a week while the product from the next stage, e.g., egg noodles, is introduced.
- If symptoms occur with a larger quantity of a new level, take a step back an try again after some time.

Step 1: Bread recipe with 1 hen’s egg

<u>Ingredients:</u>

- 500 g flour (e.g. spelt, emmer, wheat or gluten-free flour mix)
- 400 mL warm water
- 1 hen’s egg (56 g)
- 2 tsp salt
- ½ cube of yeast or packet of dry yeast

<u>Preparation:</u>

Mix the water and egg well! Mix the flour with the yeast and salt and then knead in the water-egg mixture. The dough should be rather soft; add more warm water if necessary.
Leave to rise in a warm place for ~ 30 minutes so that the dough has risen considerably. In the meantime, preheat the oven to 200 °C. Pour the dough into a greased loaf tin.
Bake the bread for 30 minutes at 200 °C, then reduce the temperature to 170 °C and bake for another 30 minutes. Allow to cool slightly, then remove from the tin and allow to cool on a wire rack.

1 loaf yields ~ 1,000 g bread = 25 slices of 40 g each; ¼ slice 10 g.
The bread is ~ 30 cm long, 1 slice is ~ 1.2 – 1.5 cm thick.

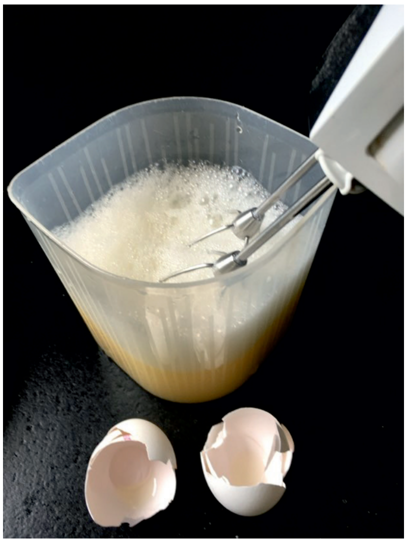

← Water and egg should be whisked into a homogeneous mass before the water-egg mixture is kneaded with the flour.

↓ Petra Funk-Wentzel. Breads, whether baked with one or two eggs, can be easily sliced and portioned into small pieces after they have cooled completely.

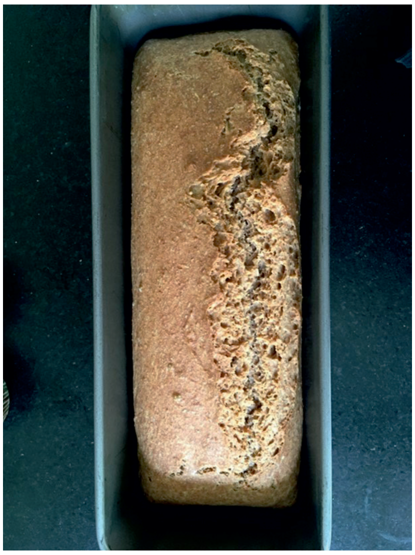

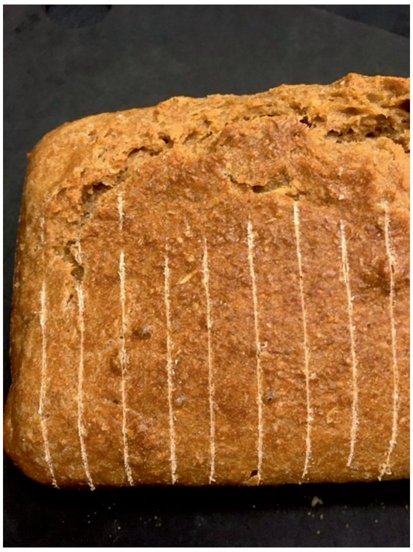

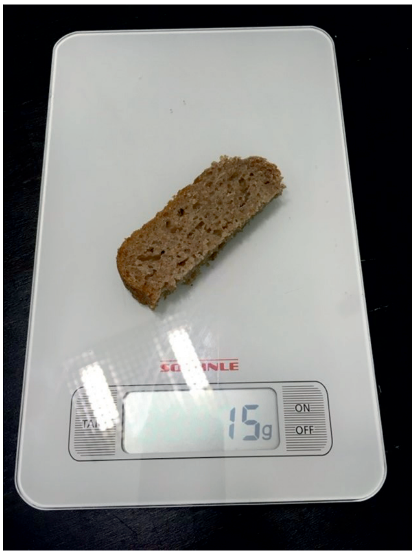

Bread recipe with 2 hen’s eggs

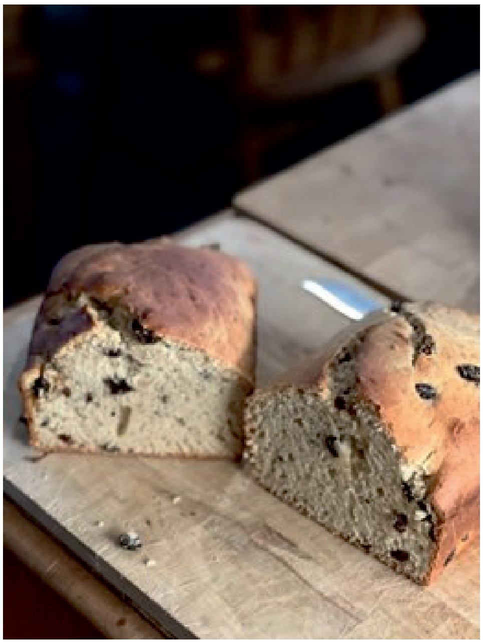

Amely Brückner. For more variety, olives, dried tomatoes or even dried fruit can be baked in.

Ingredients:

- 500 g flour (e.g. spelt, emmer, wheat or gluten-free flour mix)
- 350 mL warm water
- 2 hen’s eggs (56 g each)
- 2 tsp salt
- ½ cube of yeast or packet of dry yeast

Preparation:
Mix the water and eggs well! Mix the flour with the yeast and salt and then knead in the water-egg mixture. The dough should be rather soft; add more warm water if necessary.
Leave to rise in a warm place for ~ 30 minutes so that the dough has risen considerably. In the meantime, preheat the oven to 200 °C. Pour the dough into a greased loaf tin.
Bake the bread for 30 minutes at 200 °C, then reduce the temperature to 170 °C and bake for another 30 minutes. Allow to cool slightly, then remove from the tin and allow to cool on a wire rack.

1 loaf yields ~ 1,000 g of bread = 25 slices of ~ 40 g each; ¼ slice ~ 10 g.
The bread is ~ 30 cm long, 1 slice is ~ 1.2 – 1.5 cm thick.

Muffins with 1 hen’s egg

Ingredients for 12 muffins:

- 250 g flour (e.g. spelt, emmer, wheat or gluten-free flour mix)
- 120 g sugar or 1 large, ripe, mashed banana
- 1 pinch each of cinnamon and salt,
- 1 teaspoon baking powder
- 1/8 L cow’s milk or plant-based drink
- 1 hen’s egg (size M)
- 60 mL cooking oil
- ¼ scraped vanilla pod

Preparation:
Preheat the oven to 180 °C. Mix the dry ingredients (flour, sugar, salt, baking powder) together. In a second bowl, beat the milk, oil, vanilla pulp and egg until frothy. Gradually stir in the flour mixture. Line a large muffin tray with 12 cases and fill with dough. Bake for 30 – 35 minutes until golden brown.

1 muffin weighs ~ 45 g after baking (with ~ 10% water loss)

For savory muffins, replace the sugar (and cinnamon) with 40 g diced ham and 40 g vegetables.

Important: Use a toothpick to check that the muffins have been baked long enough. There should be no dough sticking to the toothpick. The baking time can vary from oven to oven.

Muffin recipe with 2 eggs

<u>Ingredients for 12 muffins:</u>
- 250 g flour (if necessary, use gluten-free flour, gluten-free baking mix, note: watch out for other allergens!)
- 120 g sugar or a large, ripe, mashed banana
- 1 pinch each of cinnamon and salt,
- 1/2 teaspoon baking powder
- 1/8 L cow’s milk or plant-based drink
- 2 hen’s eggs (size M)
- 50 mL cooking oil
- ¼ scraped vanilla pod

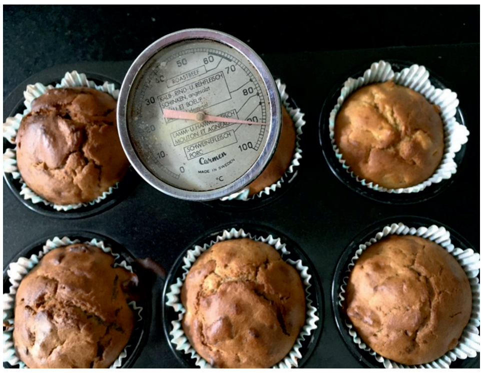

Petra Funk-Wentzel. A meat thermometer is a good way to check whether the core temperature is high enough and whether the muffins and therefore the hen’s egg are well baked.

<u>Preparation:</u>
Preheat the oven to 180 °C. Mix the dry ingredients (flour, sugar, salt, baking powder) together. In a second bowl, beat the milk, oil, vanilla pulp and eggs until frothy. Gradually stir in the flour mixture. A large muffin tray Line the oven with 12 muffin cases and fill with batter. Bake for 30 – 35 minutes until golden brown.

1 muffin weighs ~ 50 g after baking (with ~ 10% water loss)

For savory muffins, replace the sugar and cinnamon with ~ 40 g diced ham and 40 g vegetables.

<u>Important:</u> Use a toothpick to check that the muffins have been baked long enough. There should be no dough sticking to the toothpick. The baking time can vary from oven to oven.

<u>Tips:</u>
- The muffins are easier to remove from a silicone baking pan.
- The muffins freeze and thaw well. If necessary, you can cut the muffins into slices, toast them and add toppings/spreads after thawing.

Level 2 – Noodles/dried pasta with fresh egg 10%

e.g., croissants, letters, spaghetti, vermicelli, tagliatelle, etc.;

<u>Preparation:</u>
Cook pasta in boiling salted water according to package instructions.

Level 3 – Pancakes

Ingredients for 6 pieces:
- 1 egg (size M)
- 1 pinch of salt
- 125 g wheat flour, type 405 (replace with gluten-free flour mix if necessary)
- 1 pinch of baking powder
- 200 mL cow's milk or plant-based drink
- 50 mL carbonated mineral water
- Oil for frying

Preparation:
Whisk the egg and salt in a bowl. Gradually add the flour mixed with the baking powder, the milk and finally the mineral water to form a smooth dough. Leave to stand for ~ 15 minutes. Cook six equal-sized pancakes over medium heat for at least 3 – 4 minutes on each side until golden brown.

Tip: To get six pancakes of the same size, it may be helpful the first time to divide the batter into six cups and then bake these portions in the pan.

Commercial waffles are also allowed at this level. A list of these well-cooked egg-containing foods can be found following the recipes.

Level 4

At this level, you can choose between meatballs (with 1 egg) and vegetable patties (with 3 eggs). The amount of hen's eggs in the vegetable patties is three times as large as in the meatballs, so please start with much smaller consumption amounts (see hen's egg ladder graphic)!

Meatballs with 1 egg as a binding agent

Ingredients for 12 pieces:
- 1 bread roll from the previous day (~ 100 g)
- 1 (50 g) onion
- 500 g ground beef
- 1 hen's egg (size M)
- 1 tsp mustard
- 1 short tsp (3 g) salt
- Oil for frying

Preparation:
Soak the bread roll in plenty of water for ~ 30 minutes. Peel the onion and dice it very finely. Wring the bread roll well. Knead the minced meat, bread roll, egg, diced onion, mustard and salt thoroughly in a bowl. Form 12 meatballs with moistened hands.
Fry the meatballs in a pan with a little oil over medium heat until golden brown. To ensure that the meatballs are cooked through, flatten them slightly while frying and cook for at least 5 – 6 minutes on each side. If necessary, finish cooking with the lid closed or in the oven at 180 °C for a few minutes. Drain on a kitchen towel.

Vegetable patties with 3 eggs as a binding agent

Ingredients for 12 pieces:

- 150 g buckwheat or Ebly (= sun wheat), alternatively: sunflower seeds
- 2 (120 g) carrots
- 1 (150 g) courgette
- 1 (50 g) onion
- 2 tbsp (20 g olive oil)
- Salt
- 1 tbsp chopped parsley if needed
- 3 hen’s eggs
- 150 g breadcrumbs
- Oil for frying

Preparation:

Cook the beech wheat or Ebly according to the instructions on the packet, drain and allow to cool. Wash and clean the carrots and zucchini and grate finely. Peel the onion and dice very finely.

Add olive oil to the pan and sauté the onions until translucent. Add the vegetables and sauté everything for another 5 minutes. Add the vegetable mixture to the Ebly/Buckwheat in the bowl and allow to cool slightly. Season with salt and herbs if necessary. Whisk the eggs and mix in evenly with the breadcrumbs. With wet hands, form 12 flat patties. Heat a little oil in a pan and cook the patties well on each side over a medium heat until golden brown. If necessary, continue cooking with the lid closed or in the oven at 180 °C for a few minutes. Drain on a kitchen towel.

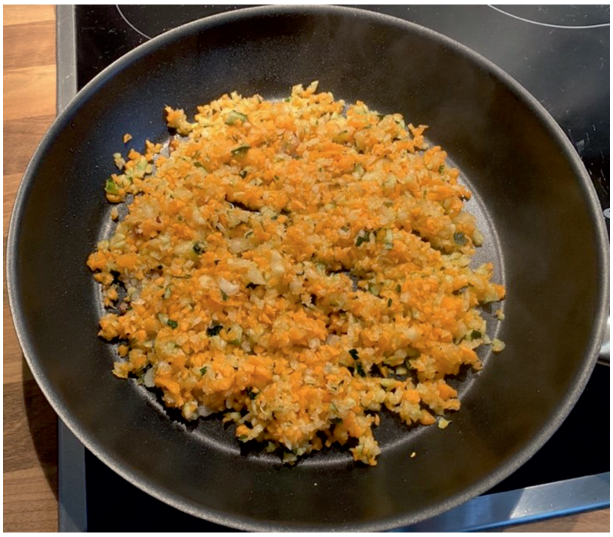

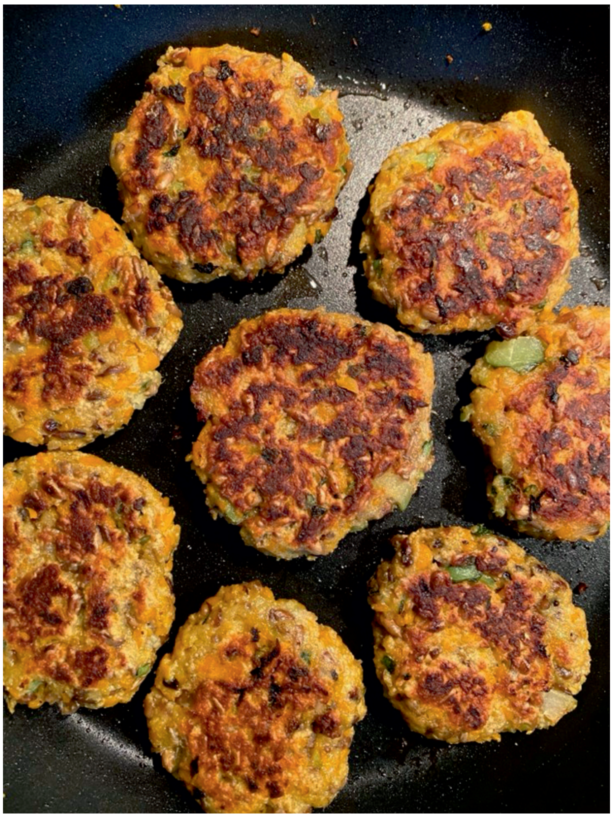

Stephanie Hompes. Vegetable patties are a tasty, plant-based alternative. It is important to ensure that they are cooked thoroughly (the hen’s egg they contain). If sunflower seeds are used instead of grains, they should be lightly roasted in a pan without fat before further processing.

Appendix III – Using the Hen's egg ladder with commercial baked goods containing eggs:

Example 1 – Continental Bakeries Ladyfingers → Recommendation Level 1

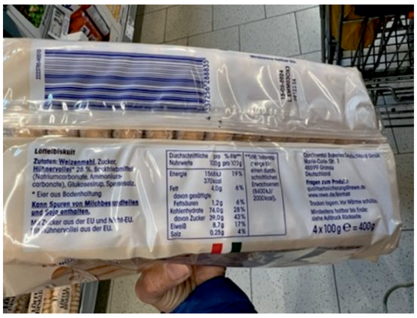

Amely Brückner/2024.

With whole hen's eggs (i.e., egg white and egg yolk) of 28%, third on the ingredients list behind wheat flour and sugar.

Hen's egg content 28 g/100 g.
There are 12 ladyfingers per 100 g. 1 ladyfinger therefore contains 0.31 g of egg protein.
This corresponds to ~ 1/2 home-baked muffin (muffin recipe with 1 hen's egg, see above) or ¼ home-baked muffin (muffin recipe with 2 hen's eggs, see above).

Similar products are also available from other suppliers (see Appendix IV).

Example 2 – ja! Egg cookies → recommendation level 1

With whole hen's eggs (i.e., egg white and egg yolk) of 24%, third on the ingredients list behind wheat flour and sugar.

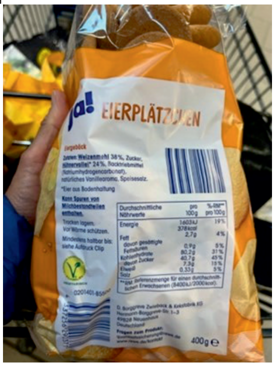
Amely Brückner/2024.

Trace information (if applicable) for milk components.

Hen's egg content: 24 g/100 g.
Each egg cookie (weighs about 8 g) contains 0.25 g of egg protein.
This corresponds to about 1 slice of homemade bread (baked with 1 hen's egg, see above) or ½ slice of homemade bread (baked with 2 hen's eggs, see above).

Example 3 – Madeleines longues by Pascal & Ernest (P&E) (made in France) → Recommendation level 3

With hen's egg (without reference to whole egg) of 23% on the ingredients list in second place behind wheat flour and before sugar.

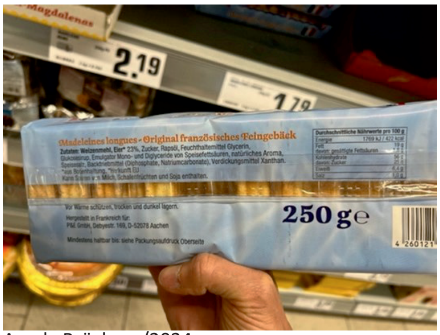

Amely Brückner/2024.

Trace information (if applicable) for milk components.

Hen's egg content: 23 g/100 g.
There are 16 madeleines in the pack.
Each Madeleine (weighs just under 17 g) contains 0.47 g of egg protein.
This corresponds to ~ 1/3 of a home-made muffin (baked with 2 hen's eggs, see above).

Caution is advised because the hen's egg is in second place in the ingredients list. The proportion of hen's eggs is high, so the Madeleines should only be consumed on the third level of the hen's egg ladder!

Example 4 – Rewe/ja! fresh egg waffles with 32% hen's egg → recommendation level 3

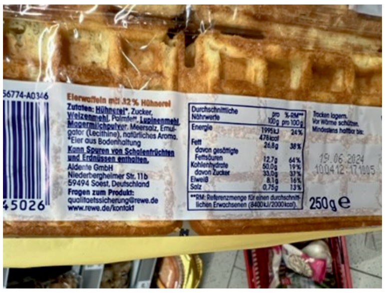

Amely Brückner/2024.

The pack contains 250 g of waffles, divided into ~ 8 smaller waffle pieces, i.e., 1 waffle weighs just under 32 g.

Trace information (if applicable) for nuts and peanuts

Hen's egg content: 32 g/100 g.
Each waffle (weighs ~ 32 g) contains 1.3 g of egg protein.
¼ waffle contains 0.33 g of egg protein. This corresponds to ~ ½ homemade muffin with 1 egg (see recipe above) or ¼ homemade muffin with 2 eggs (see recipe above).

Caution is advised because the hen's egg is at the top of the ingredients list. The proportion of hen's eggs is high, so the waffles should only be consumed on the third level of the hen's egg ladder!
In addition, other allergens (e.g., lupin flour) could be contained in the waffles (see Appendix IV)!

Appendix IV – List of commercial products containing hen's eggs

Products	Protein content/100 g	Hen's egg content/100 g	Hen's egg protein/100 g (approx.)	Information on allergens	Consump-tion in Level:
Griesson – de Beukelaer TUC Crackers Classic	8.0 g	1.3 g	0.16 g	Contains wheat, sodium sulphite, salt (1 g/100 g)<br>Whole egg in 5th place on the ingredients list<br>Trace labelling: milk components	1
Pascal & Ernest (P&E) Belgian Waffles	5.3 g	4 g	0.52 g	Contains wheat<br>Hen's egg in 11th place on the ingredient list<br>Trace labeling: milk, soy	1
Continental Bakeries French Ladyfingers	6.4 g	18 g	2.5 g	Contains wheat	1
St Michel Madeleine's Moelleues Bio	5.8 g	20 g	2.5 g	Contains wheat<br>Vanilla flavoring contains alcohol	1
Coppenrath Egg Cookies	8.2 g	24 g	3 g	Contains wheat, soy flour<br>Trace identification: milk, nuts	1
ja! Egg cookies	7.3 g	24 g	3 g	Contains wheat<br>Trace labelling: milk components	1
Linea natura spelled ladyfingers	8.5 g	26 g	3.5 g	Contains spelt flour<br>Trace labeling: milk, soy	1
Continental Bakeries Ladyfingers for Tiramisu	8.5 g	26 g	3.5 g	Contains wheat<br>Trace labelling: milk components, soya	1
Alnatura spelt spoon biscuits	11 g	29 g	4 g	Contains spelt flour<br>without sugar sprinkles	1
ja! Thread Fresh Egg Noodles	14 g	15 g	2 g	Contains durum wheat semolina	2
Birkel fresh egg noodles (e.g., croissants, letters, vermicelli, etc.)	13 g	10 g	1 g	Contains durum wheat semolina<br>Trace labeling: Soya	2
REWE Grated Spaetzle	15 g	20 g	2.5 g	Contains durum wheat semolina<br>Trace labeling: Soya	2
Rewe organic spelt fresh egg waffles/Alnatura spelt egg waffles/ja! egg waffles	8.8 g	32 g	4 g	Contains spelt, lupin flour, skimmed milk powder<br>Trace identification: Nuts, peanuts	3
Pascal & Ernest (P&E) Madeleines longues	6.4 g	23 g	3 g	Contains wheat<br>Trace labelling: milk, nuts, soya<br>Attention : Eggs in 2nd place on the ingredients list	3
linea natura spelt egg biscuits	11 g	38 g	5 g	Contains wheat<br>Trace labelling: Nuts, milk, soy<br>Attention : Eggs in 2nd place on the ingredients list	3
Schulte Biscuit Tongues	11 g	38 g	5 g	Contains wheat<br>Trace labelling: Nuts, milk, soy<br>Attention : Eggs in 2nd place on the ingredients list	3

The selection was compiled in 6/2024 according to information on the products or information from the manufacturers by Amely Brückner, Petra Funk-Wentzel, Stephanie Hompes, information without guarantee. Changes in the recipes are possible!
